# Prolonged Shower-Baths in the Treatment of the Insane
*A Letter to the Committee of Visitors of the Surrey Lunatic Asylum, by Charles Snape, Medical Superintendent (male department), in reference to the Case of Daniel Dolley (deceased).—London : John Churchill.


**Published:** 1857-01-01

**Authors:** 


### THE JOURNAL
OF
### PSYCHOLOGICAL MEDICINE
AND
### MENTAL PATHOLOGY.

JANUARY 1, 1857.

### Part First.

### Original Communications.

### ART. I.—PROLONGED SHOWER-BATHS IN THE TREATMENT OF THE INSANE.*

BY THE EDITOR.

OUR readers will remember that the Commissioners in Lunacy instituted a prosecution against Mr. Charles Snape for having, as they alleged, caused the death, on the 9th of April, 1856, of a pauper patient of the name of Daniel Dolley, confined under that gentleman's care, in the male department of the Surrey County Lunatic Asylum, by subjecting him to a continuous shower-bath of *thirty* minutes' duration, and for having administered to the said Daniel Dolley, soon after his removal from the bath, and whilst in a state of vital depression, a dose of "white coloured mixture," alleged to have contained two grains of tartar-emetic. In the last number of this Journal we intimated that the grand-jury had thrown out the bill of indictment.

Pending the trial, the Committee of the Surrey County Asylum very properly suspended Mr. Snape, appointing Dr. French, *ad interim*, to superintend the male side of this institution. It will gratify the friends of Mr. Snape to hear that he has been reinstated into office. Six medical gentlemen of position, character, and reputation were selected to decide the question of re-nomination. Three were appointed by the Committee

* A Letter to the Committee of Visitors of the Surrey Lunatic Asylum, by Charles Snape, Medical Superintendent (male department), in reference to the Case of Daniel Dolley (deceased).—London: John Churchill.

of the Asylum, and three were named by Mr. Snape himself. We believe these gentlemen were unanimous in recommending that Mr. Snape should be restored to his former official position. Such being the verdict of the professional jury delegated with authority to settle the question of re-appointment, we are in a measure precluded from discussing the important points involved in the bill of indictment against Mr. Snape.

It would be obviously unfair to this gentleman to re-open the question. He should have the full benefit of his entire acquittal at the hands of two tribunals, who adjudicated upon his case. Here the matter should and ought to have rested, if Mr. Snape had been well advised. Considering himself much aggrieved, his character to have been seriously reflected upon, his skill and humanity to be gravely questioned, Mr. Snape might with propriety have defended himself against the specific offence imputed to him, *viz.*, that of causing the death of Daniel Dolley by wilful negligence, unskilful and unscientific treatment. No one would or could have blamed him for so reasonable and natural an act of self-justification; but the question assumes altogether another and a serious aspect, when a formal and studied argument is put forward in defence, not of his treatment of this particular case, but of prolonged shower-baths, as a safe, efficient, judicious, and curative process of treatment.*

Such being the position taken by Mr. Snape, in the pamphlet which he has published, we have no alternative but to pause and

* Our non-professional readers should remember that we are not now speaking of an ordinary harmless *domestic* shower-bath, but one of formidable dimensions. The following extract from the report made for the Commissioners in Lunacy by Mr. C. Vignoles, civil engineer, will convey some idea of the size of the bath used in the Surrey County Lunatic Asylum, and which Mr. Snape alleges he has been using for four or five years:—"On the supposition that the supply was equal to the discharge (during the time the lunatic remained in the shower-bath) after the water had fallen to the minimum head of four inches, the following must have been the quantity of water discharged upon him during the above twenty-eight minutes, viz :—

| | | | | |
|---|---|---|---|---|
| In the first half-minute . . | 19½ gallons, | or 39 gallons per minute. | | |
| In the next minute and a-half | 39 ditto | or 26 | ditto | |
| In the last 26 minutes . . | 507 ditto | or 19½ | ditto | |
| | 567 gallons in 28 minutes, | | | |

being an average discharge equal to the contents of a *twenty-gallon-cask of water per minute* for nearly half an hour upon the lunatic." Mr. Snape, questioning the accuracy of the above calculations, appointed his own engineer to examine the capacity of the bath, and, in justice to the accused party, we append his view of the matter in dispute :—

"The total contents of the cistern when full are *not* 400 gallons, as stated by Dr. Diamond, but 91 gallons ; and the total quantity discharged during twenty-eight minutes, by actual test for that period, and not by estimate—was not 618 gallons, as stated by Mr. Shields, but 477 gallons; of which *not more than* 119 *gallons* could have passed over the body of Dolley *during the twenty-eight minutes,* the remainder not touching him ; while even of the 119 gallons, it is evident that the larger portion must have been wholly turned off from his body by his two hands, which he placed over his head."

consider these questions,—Is Mr. Snape right in his view of the treatment of the insane, and has he, as he appears to believe, discovered a new and valuable agent for the cure of Insanity? This subject cannot be ignored. It comes formally and legitimately before us; Mr. Snape has thrown down the glove, and challenged the profession to the combat, and we should be guilty of a serious dereliction of duty as public journalists, if we, out of any feelings of false delicacy, were not to enter the arena, and subject the point at issue to a close and rigid examination. If Mr. Snape is right, then he should have all the credit due for suggesting a novel and beneficial mode of treating one of the most distressing class of affections with which the medical man has to deal. If, on the other hand, we consider the use of the prolonged shower-baths, with or without large doses of tartrate of antimony, positively mischievous and unjustifiable, we should be criminal if we were to be silent on the subject. We repeat, that we are *forced* into this inquiry; Mr. Snape's published defence of his treatment makes it imperative. In considering the question before us we will, as far as practicable, leave the particular case of Daniel Dolley entirely out of discussion.

It would appear from Mr. Snape's statement that the prolonged shower-bath, as a remedial mode of treatment in cases of insanity, is no new-fangled or suddenly conceived notion, which has just sprung into mental existence, but that he has, on the inductive method, *for years* been carefully trying experiments with the baths, and has assured himself of their efficacy and safety. Such is the conclusion clearly deducible from the subjoined paragraph:—

"Why visit with the utmost denunciation of the law, and subject to the obloquy and ruinous consequences of a public prosecution as a felon, a medical man elected on account of his past experience and medical attainments to such a post, because he has the courage *to carry onward remedial measures which, for a period of four or five years, he has been gradually applying, and with unvarying success;* although to other institutions even the treatment so long practised by him may be unknown, and, therefore, may be too hastily and most erroneously regarded, *speaking from* THEORY *only*, as dangerous and bad?"

We think it would have been more discreet if Mr. Snape had omitted all reference to the treatment pursued in other Asylums, and had confined his observations to the defence of his own peculiar practice. When speaking of the uncertainty of medical treatment in all cases of disease as a justification, we presume for his own happy "guess," Mr. Snape remarks:—

"The science of medicine in all its branches will ever be a science of unusual difficulty and doubt, from the necessity of treatment being based upon 'surmise;' and, after all, he is the ablest practitioner who guesses most correctly, and assumes most justly the real seat and nature of the disease to be grappled with. But it is still 'surmise;' and if in the treatment of bodily ailments there be so much doubt, how much greater is the doubt in reference to mental disease!"

We enter our protest against this singularly inaccurate and unhappy statement. Mr. Snape is not justified in representing the science of medicine to be "based upon *surmise*," or in asserting that he is "the ablest practitioner who GUESSES most correctly, and ASSUMES most justly, the real seat and nature of the disorder to be grappled with." The science of medicine is not open to so serious and fatal an imputation. If Mr. Snape had contented himself with repeating the old hacknied assertion, that medicine was an *uncertain* science, we should not have objected to the dogma. And why is the science of medicine an "uncertain" one? Not because the medical practitioner has to "guess," "assume," and "surmise," as Mr. Snape represents, but because he has to deal with an organic *living* machine—with a body endowed with *mind*, and with *life*—subtle principles, of the nature of which we know little or nothing—principles of *vitality* and *intelligence* always in operation, modifying the character of healthy and morbid action, and powerfully influencing the *modus operandi* of remedial agents exhibited for the cure of disease.

It is a libel upon the medical profession to say that the treatment of disease is "based upon SURMISE" and "GUESSES," or that "he is the ablest practitioner, who GUESSES most correctly." This is the language of the empiric, not the man of science. If a patient were brought to the Surrey County Lunatic Asylum, suffering from *delirium-tremens*, would not Mr. Snape's experienced eye immediately recognise the malady? There would be no "guessing" in the matter; he would, without hesitation, designate the disease by its right name. Again, when called upon to grapple with the case remedially, his treatment would not be based upon "*surmise*," neither would he "guess" at his therapeutic agents. Instinctively he would seize upon the appropriate specific. In the treatment of the ordinary diseases which come under the notice of the physician in every-day practice, such as gout, pneumonia, enteritis, gastritis, as well as in the numerous class of cutaneous affections, where is the "guessing," the "assuming," and the "surmising"? Surely there are intelligent and well-educated men in the profession, who can diagnose with extraordinary accuracy the pathological character and seat of the principal morbid changes that may have taken place in the various vital and organic tissues? Consider, for example, the organ of respiration. In the affections of this organ, is not the experienced physician fully competent to pronounce authoritatively upon the case, as one either of congestion, inflammation, or hepatization? Is not the existence of pulmonary tubercles easily detected? Is it not possible to say with certainty whether a cavity does or does not exist in the lungs? This is not "guessing" or "surmising." So wonderful is our existing knowledge of the characteristics and pathology of disease, that it is rare for a well-educated and experienced practitioner to commit an error, either in his diagnosis or treatment. He does not, unhappily, always succeed in curing his patient, but this does not arise from a wrong "guess," or an unlucky "surmise," or unfortunate "assumption," as Mr. Snape would lead the public to believe. The profession of medicine would indeed be reduced to a very low ebb, and the practitioner of this exalted art would be in a humiliating position, if the noble science which he cultivates and practises were based upon "*guesses*," "*assumptions*," and "*surmises*." There is much in pathological science still *sub-judice;* and there are a few diseases which continue to baffle the physician, and set at defiance the best directed of his most powerful remedial agents. This, necessarily, must always be the case; but this generally acknowledged fact does not justify the sweeping and indiscriminate charge brought by Mr. Snape against the science of medicine. God forbid that the physician who, in the language of Mr. Snape, "guesses most correctly, and assumes most justly," should ever be considered as "the ablest practitioner."

But to proceed. Mr. Snape quotes approvingly from a recent work of Dr. Conolly, who, when speaking of the "obscurity" of our "pathology of mental disorders," observes, "there is still no reason to abandon the hope that fresh resources will some day be possessed by the practitioner, and that the real nature of the changes taking place in the brain may be better understood, and greater success attend medical treatment." We cordially re-echo the wish expressed by Dr. Conolly; but in what way do these remarks bear upon Mr. Snape's defence of prolonged shower-baths? He alleges that he ought not to be "denounced as a barbarous practitioner for advocating a course of treatment which he has practically proved to be so good and valuable in itself (speaking of twenty minutes' continuous shower-baths), because others who have *not tried* this treatment *consider* it as dangerous and unsafe." Certainly not. It would be monstrous to condemn a man on such grounds. But he must first clearly establish that he has discovered a course of treatment not only "good and valuable in itself," but safe and judicious. If Mr. Snape considers that the "prolonged shower-baths" are the "fresh resources" in the way of treatment, which Dr. Conolly hopes may at no distant period dawn upon the psychological horizon, then we are bound honestly to ask ourselves these questions, are they *remedial* in their effects upon the insane; and are they *safe* modes of treatment? Mr. Snape alleges, that he speaks not only of the efficacy, but of the safety of these baths, basing his observation and conclusion upon "four or five years' most valuable experience." He says:—

"I have been in the frequent habit of administering continuous cold shower-baths to insane patients for periods of fifteen and twenty minutes, with and without intermissions of a few seconds, with the greatest success. I NEVER KNEW THE SLIGHTEST ILL RESULT, and instances can be given of *entire restoration to reason by one single fifteen or twenty minutes' continuous bath:* added to which, there are cases, which I should have proved had my case gone to trial, in which discharged patients have imputed their restoration solely to these long baths."

This is strong evidence in favour of Mr. Snape's treatment, and it is entitled to our respectful consideration. Such being the result of his "*four* or *five* years' valuable experience," we think he should have placed upon record, in the pamphlet under consideration, a more satisfactory and scientific classification of the cases in which he has used, with advantage and safety, the prolonged shower-bath, and have, at the same time, clearly expounded to his professional brethren a sound *principle* of treatment, as well as described the forms of insanity for the cure of which this remedy is adapted. Without *data* of this kind no general assertion of the curative efficacy of any particular course of treatment can meet with the deference and attention of scientific men. Are the prolonged shower-baths safe and useful in the treatment of acute mania, monomania, melancholia (acute and chronic), dementia, general paralysis? Upon these important points Mr. Snape throws no light:

"He dies and makes no sign."

He uses the general phrase, "Insanity," forgetting that this term conveys but a vague and unsatisfactory idea to the mind of the pathologist when called upon to estimate the value of any special course of medical treatment. A patient is brought to the Asylum in an acute state of mania, verging upon cerebritis, exhibiting all the well-recognised symptoms of sanguineous congestion of the brain. The patient is wildly delirious—the scalp hot—the skin dry and parched—the conjunctivæ injected—the pulse rapid: in common parlance, he is insane. Is this, we ask Mr. Snape, a case for the prolonged shower-bath of fifteen, twenty, or thirty minutes' duration? Again, a man enters the hospital so physically reduced as to be unable to creep into the wards. He has the physiognomy of an imbecile; he cannot articulate; he falters and staggers in his gait; his general bodily condition is that of extreme prostration. Associated with these symptoms, his mind is in a morbid state of exaltation. He asserts that he is the possessor of a fabulous amount of wealth, and maintains that he is a king, an emperor, or our Saviour: in other words, he is suffering from the general paralysis of the insane. Is this a type of case in which Mr. Snape would advise the use of the prolonged shower-bath? Without something more specific from the pen of this gentleman, his general, wholesale, and indiscriminate recommendation of this remedial agent in the treatment of insanity is worse than valueless. But Mr. Snape not only believes that he has succeeded in "guessing" at the "fresh resource" spoken of by Dr. Conolly as the therapeutic desideratum, but that the reason why it has been so ignored and condemned, is, that the professional mind is not yet sufficiently matured and ripe to receive the great truth which he has had the good fortune to discover, and the moral courage to propound. *Credat Judæus?* In other words, this wonderful and happy "guess" of prolonged shower-baths, is only second in importance to the discovery of railway travelling!

Mr. Snape remarks—

"When the late Mr. George Stephenson was asked by a Commons railway committee, in the year 1836, whether a railway train could travel at the rate of a mile a minute without danger, his sagacious reply was 'Yes; *but the public mind is not yet prepared to receive that truth as a fact.*' Such is precisely the present state of the 'shower-bath' question."

Fustian and bombast! Surely Mr. Snape is a wag, and has put this forward as a piece of pleasantry? After reading the paragraph,

"*To be grave exceeds all power of face.*"

Mr. Snape would wish the public and profession to believe that he is another Galileo—a second Jenner: prosecuted, persecuted, hunted down, condemned, tabooed, and vilified, because he has had the good fortune to make a *discovery considerably in advance of the age!*

"*Ye lesser stars, hide your diminished heads,*"

and prostrate yourselves before this modern psychological luminary. When a man commits so egregious an act of folly as to go about the town upon a pair of stilts, with a ticket tied to the tail of his coat, on which is inscribed in large and legible characters the word *Martyr*, it is time that his ostentatious pretensions to so honourable a designation, and to so high a distinction, should be freely, fully, and unreservedly canvassed and criticized. Mr. Snape has only himself to blame if he does not come out of the field with a flourish of trumpets and flying colours.*

* One would imagine, from the way in which Mr. Snape writes, that he was entitled to all the credit of having been the first practitioner who advised the use of

Feeling himself to be in a difficulty as to the best mode of justifying his use of the prolonged shower-baths—appreciating the necessity of expounding some *principle* of treatment in connexion with them, Mr. Snape again shields himself behind the back of Dr. Conolly; but, conscious of the weakness of his case, he summons two other physicians to his support. He waves his magic wand, and *presto*, Drs. Elliotson and Davey appear upon the scene. And what do our readers conceive to have been the *principle* which has guided Mr. Snape in the treatment of the insane, and which, according to his mode of reasoning, justified the use of the prolonged shower-baths? Before proceeding further in this part of our inquiry, we would premise that in no science, so much as in medicine, is a reference to principles of so much consequence, for nowhere does the mere sequence of events, the *post hoc propter hoc* mode of argument, so often lead into error.

"Without principles," says the great Dr. Cullen, "deduced from analytical reasoning, experience is a useless and a blind guide." Appreciating this truth, Mr. Snape protects himself—in fact, hedges himself in—by enunciating to the scientific world the principle which has led him through the obscure and hazy paths of mental pathology to the great fountain, source and light of all sound therapeutic knowledge. "What," says Mr. Snape, "is the purpose for which the shower-bath is recommended?" The object of the practitioner (quoting approvingly the words of Drs. Conolly,* Davey, and Elliotson), in the administration of the the shower-bath in the treatment of the insane! As far back as the time of Boerhaave they were used in cases of insanity; and every modern writer on the subject speaks of their efficacy in certain forms of mental derangement. A physician is in the habit of prescribing six or ten grain doses of *quinine* in cases of intermittent fever. His neighbour ventures, under similar circumstances, to administer half-drachm doses of the same drug. If the dose were considered safe and judicious, and were in a few instances to succeed in stopping the progress of the fever, no person outside of St. Luke's would think of placing this practitioner upon a pedestal, or consider him a Jenner, or a John Hunter; neither would his friends, when he passed to the tomb of all the Capulets, have the courage to apply to Parliament for a public grant to erect a statue to his memory! Admitting Mr. Snape to have had the good fortune to hit upon an efficient remedy, what is really his due? It is perfectly preposterous for him to talk of his having made any discovery at all; and still more ridiculous for him to assert that his discovery is in advance of the age!

* Dr. Conolly never recommended continuous shower-baths of any considerable duration. He speaks favourably of the effects of "*intermittent* shower-baths," and says, "if employed *in the ordinary manner* (that is, we presume, for *three* or *four* minutes), its effects are rather exciting than depressing." Mr. Snape would lead us to believe that intermittent baths are more *distressing* and dangerous than a *continuous* shower-bath! How can this be? A patient who is subjected to a shower-bath of ten or fifteen minutes' length, with a clear intermission of two or three minutes after the expiration of every four or five minutes' bath, must, of necessity, have a much less shock than he would be subjected to if the bath were continuous for a period of fifteen or twenty minutes. In the intermittent baths, the patient is permitted occasionally to rally his powers of life; this cannot occur to the man who has a continuous bath.

shower-bath, is the "*overpowering of the patient,*" "*the prostration of the system;*" it is to be continued "*until decided prostration ensues.*"

If such is the principle upon which Mr. Snape regulates his administration of the prolonged shower-bath, or, in fact, any other of his remedial agents, lamentable, indeed, must be the result to the unhappy patients subjected to the treatment. This may be considered strong language, but we cannot finesse, or use equivocal and ambiguous terms, when discussing a matter involving not only the reason but the lives of so many of our afflicted fellow-creatures. We do not hesitate to assert that the principle of treatment propounded by Mr. Snape is glaringly inconsistent with the modern and approved views of the pathology of insanity. The practice of "overpowering" an insane patient, of "prostrating the system," of pursuing any plan of treatment, "until decided prostration ensues," is indefensible and unjustifiable.

In insanity, the *vis vitæ* is often reduced to the lowest possible condition. In the great mass of acute cases of disordered mind which the physician is called upon to treat—particularly in our public Asylums—the nervous system is in a state of positive exhaustion and debility. The furor,—the violence,—the maniacal excitement; the muscular resistance, so often associated with insanity, are generally symptomatic of *profound nervous and vascular depression.* The excitement of the insane is an *excitement without power*, and he is truly the "ablest physician" who, recognising this important pathological fact, does his utmost *to conserve and husband the flagging and ebbing vitality of his patient,* until the mind recovers its healthy equilibrium.

Insanity does not result from *active* inflammation of the brain, and, if such were its origin, no physician would be justified in attempting to "*prostrate the system*" of those mentally disordered. In cases of profound cerebral excitement, the patient often recovers under the combined influence of a *tonic and stimulating* plan of treatment. We have known violent mental perturbation considerably mitigated, and often cured, by the administration of wine and stout combined with iron and quinine. To put a patient, because he is "violent," "noisy," "excited," and "destructive," into a shower-bath of fifteen or twenty minutes' duration, with a view of "*prostrating*" his energies, is a practice that cannot be too gravely and seriously censured.

The physician may, by this mode of treatment, make a violent, excited, and unmanageable patient quiet and docile for a time; *but it will be quietness and docility gained at the expense of his reason, and perhaps of his life.* We are not called upon to mince words, or delicately and nicely weigh the import of phrases, when addressing ourselves to a point of such practical magnitude and importance. Facts—an overwhelming mass of evidence—conclusively, irresistibly, and incontrovertibly establish, beyond all doubt, *that the depressing, lowering, and overpowering plan of treating the insane is most disastrous in its results.* Why is the lancet never now used in cases of insanity? Why is the strictly antiphlogistic treatment so studiously ignored? Because all practical, sagacious, and experienced psychologists, having to do with the care of the insane, recognise in the morbid affections of the mind a condition of brain and nervous system which will not tolerate a "depressing," "overpowering," and "prostrating" mode of treatment. What would be the effect—the *certain* effect, if the practitioner, merely considering the conditions of violence, excitement, and mischief, in a case of *delirium-tremens*, were to subject the patient to a prolonged shower-bath? Consider, again, the consequences of a similar course of treatment in a case of puerperal insanity? In this class of cases we often witness extreme excitement and violence associated with profound vascular and nervous depression. How dangerous must then be a practice which overlooks entirely the *pathological* state of the patient! If the treatment of the insane is to consist in a rapid reduction of the physical and mental powers of the patient to the *minimum* standard; if it be all-important to make a noisy, excited, destructive lunatic tractable and quiet, so as to preclude the necessity for the use of mechanical restraint,* then continue the prolonged shower-baths, followed by large nauseating doses of tartar-emetic; but if it be the intention to carry out a *curative process of treatment*, we must, by every effort in our power, husband and conserve the vital energies until reason ascends her throne.

We have hitherto confined our observations to the effects of

* With something like a *presentiment* of the substitution of the shower-baths and *tartar-emetic* for mechanical restraint, we penned the following remarks in the October number of this Journal, for the year 1854:—"Have not the frequent administration of nauseating doses of the tartrate of antimony, the shower and cold bath, in several asylums, *taken the place of mechanical restraint, producing, as can be readily conceived by those conversant with the pathology of insanity, the most disastrous consequences?* The use of the milder forms of mechanical restraint in cases of acute and dangerous insanity can do little or no permanent injury; but the repeated and continuous exhibition of tarter-emetic, chloroform, and stupifying doses of opium, with the view of subduing the muscular violence of the insane, and thus reducing them to a manageable condition, and obviating the necessity for mechanical restraint, *may do serious and irremediable mischief;* and for this obvious reason, that the patient is compelled to take medicines which greatly depress the nervous system, at a time when everything should be done to sustain the *vis vitæ*, and give increased impetus to the nerve force. It does not require much sagacity to reduce, by these means, a violent lunatic to a state of comparative composure and quietude; but we would caution all engaged in the anxious and responsible duties of treating the insane, against the adoption of a course alike dangerous to life and perilous to reason."

the prolonged shower-bath, as the distinguishing feature in Mr. Snape's practice. Let us now briefly direct the attention of our readers to its necessary adjunct. It appears that Mr. Snape administers to his patients, after coming out of the prolonged shower-bath, one and a half to two grains of tartar-emetic! In other words, he, in addition to the shower-bath, exhibits an *emetic* to the patient. The above dose may not always induce vomiting, but it must do so in many instances. A man in health could not easily take *one* grain of the tartrate of antimony without disgorging the contents of his stomach. The insane, we admit, may take larger doses of this nauseating medicine with impunity; but, nevertheless, such a dose would cause violent vomiting even among the insane. If the medicine has not this effect, it will certainly lower and depress the powers of life, and we presume it is administered with this object. Let our readers pause for one moment to consider a patient suffering from a *depressing and exhausting disease* (and insanity is essentially one of this type), exposed to the still more depressing influence of a *continuous* fall of a large volume of water upon the head for a period of fifteen, twenty, and thirty minutes. He emerges out of his bath (as he must do) with the powers of life reduced to the minimum point. Not satisfied with this amount of physical exhaustion, the poor, helpless patient has his *vis vitæ* still further lowered by administering to him a large dose of tartar-emetic!

What is the principle involved in this plan of treatment, and why is the tartrate of antimony administered *at all* in such a state of the system? We can well understand in the early stages of sub-acute insanity, analogous in its symptoms to phrenitis, why nauseating doses of this medicine should cautiously be given; but in our judgment it is inadmissible in chronic forms of insanity, particularly when exhibited immediately after the use of a depressing prolonged shower-bath. This practice is opposed to the experience of most men engaged in the treatment of the insane, and we shall be glad to hear of its being altogether abandoned in the Surrey County Lunatic Asylum. In defence of his mode of treatment, both by the prolonged shower-bath and the tartrate of antimony, Mr. Snape has appended to his pamphlet a series of tables, illustrating his peculiar mode of practice. In justice to that gentleman we transfer to our pages the whole of this tabular statement, without abridgment or alteration. In these tables Mr. Snape cites the particulars of *fourteen* cases of alleged "cure," extending over the "*four* or *five* years," during which he has been experimenting with the bath! Is this not a small ratio of cure? viz., not ONE *per cent. per annum* out of the *four hundred male patients* on his side of the establishment always under his treatment!

### SELECTION FROM CASES

*In which Shower Baths and Tartrate of Antimony have been administered to Patients under Mr.* SNAPE'S *Care, and Extracts from Medical Case Books in support of the Treatment and its Effects.*

| No. | Age of Patient on Entry. | Length of Bath administered, in Minutes. | When followed by Tartrate of Antimony, the Quantity. | Extracts from Case Book of Special Notes in reference to Treatment. | Result to Patient, 1856. |
|---|---|---|---|---|---|
| 1 | About 60 | 15 or 20 | $1\frac{1}{2}$ and 2 grains | 29th Sept., 1841. Admitted.<br>22nd Dec., 1854. Remains unchanged; suffers occasionally from attacks of maniacal excitement, during which he is disposed to be mischievous and violent to others. *Shower-bath and tartar emetic are the remedies which have been employed.* | In Asylum. |
| 2 | 57 | 15 or 20 | $1\frac{1}{2}$ and 2 grains | 1st March, 1854. Admitted.<br>14th June, 1856. He is subject to occasional attacks of maniacal excitement, with a disposition to be violent to others. *Shower-baths and tartar emetic mixture have been found beneficial.* | In Asylum. |
| 3 | 51 | 20 | | 16th Dec., 1848. Re-admitted. | |

| No. | Age of Patient on Entry. | Length of Bath administered, in Minutes. | When followed by Tartrate of Antimony, the Quantity. | Extracts from Case Book of Special Notes in reference to Treatment. | | Result to Patient, 1856. |
|---|---|---|---|---|---|---|
| | | | | 6th June, 1853. | Occasional shower-baths have been given with good effect. | |
| 4 | 61 | 15 or 20 | $1\frac{1}{2}$ and 2 grains | 17th March, 1849. | Admitted. | |
| | | | | 9th Jan., 1854. | He continues to have paroxysms of maniacal excitement, with a disposition to violence. *An occasional shower-bath and tartar emetic mixture have been prescribed with advantage.* | |
| | | | | 20th Dec., 1855. | He continues to have periodical attacks of excitement, during which he is disposed to be violent in his conduct. *Shower-baths, with the mix. ant. tart., are sometimes found beneficial.* | |
| 5 | 40 | 15 or 20 | $1\frac{1}{2}$ every 3 hours | 4th Dec., 1851. | Admitted. | |
| | | | | 5th ,, ,, | Mix. tar. ant. 3 tablespoonfuls every 3 hours. | |
| | | | | 7th ,, ,, | Broke windows; excited; shower-bath; continue ant. potass. tart. | |
| 6 | 26 | 20 | 2 grains | 2nd Feb., 1852. | Admitted. | Cured. |

| No. | Age of Patient on Entry. | Length of Bath administered, in Minutes. | When followed by Tartrate of Antimony, the Quantity. | Extracts from Case Book of Special Notes in reference to Treatment. | | Result to Patient, 1856. |
|---|---|---|---|---|---|---|
| | | | | 4th Feb., 1852. | Conducted himself in a violent and noisy manner; shower-bath and a large dose of tart. emetic; promised betterbehaviour for the future. | |
| | | | | 21st „ „ | Made attacks upon those around him with a knife. A shower-bath and a grain and half of tart. emetic every six hours; gruel diet; to be kept under strict surveillance in No. 3 Day-room. | |
| | | | | 23rd Feb., 1855. | Is perfectly rational in his conductand conversation, and, at his urgent request, the medicine and shower-bath have been ordered to be discontinued. | |
| | | | | 28th Oct., 1853. | Broke windows. | |
| 7 | 57 | 15 or 20 | 2 grains | 3rd April, 1852. | Admitted. | Cured. |
| | | | | 7th „ „ | Ant. tart. | |
| | | | | 9th „ „ | Shower-bath. | |
| | | | | 12th „ „ | To have shower-bath every morning. | |

| No. | Age of Patient on Entry. | Length of Bath administered, in Minutes. | When followed by Tartrate of Antimony, the Quantity. | Extracts from Case Book of Special Notes in reference to Treatment. | Result to Patient, 1856. |
|---|---|---|---|---|---|
| | | | | 9th Jan., 1854. Re-admitted.<br>20th Feb., „ Abusive and destructive of his clothing, when an occasional shower-bath is given. | |
| 8 | 40 | 20 | 2 grains | 3rd May, 1852. Admitted.<br>5th May, „ Shower-bath and tart. emetic.<br>7th „ „ Shower-bath. [*N.B. This patient imputed his recovery to the shower-bath.*] | Cured. |
| 9 | 58 | 15 | 1½ or 2 grains | 20th July, 1852. Admitted.<br>15th June, 1853. He continues to labour under periodical attacks of maniacal excitement, at which times he is very noisy, abusive, threatening, and violent. *A shower-bath and dose of mixt. ant. tart. have been employed with advantage on two or three occasions.*<br>19th June, 1855. No alteration; baths occasionally. | In Asylum. |
| 10 | 28 | 20 | 1 grain | 30th July, 1852. Admitted.<br>31st „ „ Tart. Anty. | Cured. |

| No. | Age of Patient on Entry. | Length of Bath administered, in Minutes. | When followed by Tartrate of Antimony, the Quantity. | Extracts from Case Book of Special Notes in reference to Treatment. | Result to Patient, 1856. |
|---|---|---|---|---|---|
| | | | | 31st Oct., 1852. Threw down an attendant; shower-bath twice a day and seclusion; 1 grain of anty. 3 times a day. | |
| 11 | 58 | Not known | Nil | 27th Nov., 1852. Admitted.<br>5th April, 1853. *A shower-bath has been given on two or three occasions with apparent benefit.* | Cured. |
| 12 | 41 | 20 | 2 grains | 29th Nov., 1852. Admitted.<br>29th March, 1854. Has been labouring under maniacal excitement, with a disposition to be violent to others, for several weeks past. *The shower-bath, with an occasional dose of mix. tart. ant., together with purgatives, have been employed with the greatest advantage.*<br>26th Dec., „ Excitement; the same remedies have been employed with marked benefit.<br>Dec., 1855. He continues to have | In the Asylum. |

| No. | Age of Patient on Entry. | Length of Bath administered, in Minutes. | When followed by Tartrate of Antimony, the Quantity. | Extracts from Case Book of Special Notes in reference to Treatment. | | Result to Patient, 1856. |
|---|---|---|---|---|---|---|
| | | | | | occasional attacks of periodical excitement, which have been treated as heretofore. | |
| 13 | 21 | 20 | 2 grains | 16th July, 1853. | Admitted. | In the Asylum. |
| | | | | 29th Nov., „ | Bath and emetic. | |
| | | | | 10th June, 1854. | He has had two or three paroxysms of maniacal excitement since last report (22nd Dec.), *for which shower-baths and a draught containing two grains of tartar emetic have been administered with most beneficial effect.* | |
| 14 | 64 | | | 6th Aug., 1853. | Admitted. | Cured. |
| | | | | 23rd „ „ | Shower-bath every morning. | |
| 15 | 26 | 20 | Not stated | 7th Feb., 1854. | Admitted. | |
| | | | | 14th March, „ | Shower-bath ordered to be given occasionally. | |
| | | | | 10th June, „ | Ditto ditto. | |
| 16 | 37 | 20 | 4 grains | 23rd March, 1854. | Admitted. | Cured. |
| | | | | 25th May, „ | Shower-bath and emetic. | |
| | | | | 29th June, „ | Slightly excited and violent. Shower-bath twice a day, with emetic. | |

| No. | Age of Patient on Entry. | Length of Bath administered, in Minutes. | When followed by Tartrate of Antimony, the Quantity. | Extracts from Case Book of Special Notes in reference to Treatment. | | Result to Patient, 1856. |
|---|---|---|---|---|---|---|
| | | | | 10th Aug., 1854. | Repeat bath and mixture. | |
| | | | | 14th June, „ | Excitement. Shower-bath twice a day, and tartar emetic mixture, 2oz. (2 grains anty.) occasionally. | |
| | | | | 20th June, 1854. | Has become quiet and tranquil. *The shower-bath, with the tartar emetic, are remedies which appear most efficacious in subduing cerebral excitement. He is now employed in his trade.* | |
| | | | | 15th July, 1855. | Relapse. Shower-bath morning; warm bath night. Tartar emetic mixture (2 grains to a dose) occasionally. | |
| | | | | 29th „ „ | Shower-bath, warm-bath continued, with morphia. | |
| | | | | 12th Dec., „ | Remedies repeated. | |
| 17 | 34 | 20 | 1½ and 2 grains | 19th April, 1854. | Admitted. | Still in Asylum. |
| | | | | 2nd Oct., „ | Shower-bath every morning; warm bath every night. | |
| | | | | 5th Dec., „ | *Shower-bath and warm* | |

| No. | Age of Patient on Entry. | Length of Bath administered, in Minutes. | When followed by Tartrate of Antimony, the Quantity. | Extracts from Case Book of Special Notes in reference to Treatment. | Result to Patient, 1856. |
|---|---|---|---|---|---|
| | | | | *bath, with tartar emetic, are prescribed with advantage.* | |
| | | | | 25th Jan., 1855. Occasionally labours under great maniacal excitement. Shower-bath; occasionally blistering liquid to the back of neck. | |
| | | | | 5th Jan., „ Highly excited. Tartar emetic 2 oz. occasionally, and shower-bath. | |
| | | | | 10th July, „ He continues to labour under a good deal of cerebral excitement, *which appears to be much subdued by a good* STRONG *shower-bath* and warm bath, and morphia at bed-time. Castor-oil occasionally. | |
| | | | | 1st Aug., „ Much more tranquil and composed. | |
| | | | | 25th Sept., „ Excited and violent; shower-bath & tart. ant. | |
| | | | | 10th Oct., „ Remedies continued. | |
| 18 | 41 | 2 | 2 grains | 16th Jan., „ Admitted. | In the Asylum. |

| No. | Age of Patient on Entry. | Length of Bath administered, in Minutes. | When followed by Tartrate of Antimony, the Quantity. | Extracts from Case Book of Special Notes in reference to Treatment. | | Result to Patient, 1856. |
|---|---|---|---|---|---|---|
| | | | | 29th March, 1855. | Shower-bath and tart. ant. occasionally. | |
| | | | | 29th May, „ | Shower-bath every morning, and tart. ant. mix. 2 oz. (2 grs. a dose) occasionally; warm bath at night. | |
| | | | | 21st June, „ | Repeat. | |
| | | | | 16th „ „ | Repeat shower-bath and tart. emetic. | |
| | | | | 25th „ „ | Continue. | |
| 19 | 19 | 15 | Nil | 23rd Jan., 1854. | Admitted. | Cured. |
| | | | | 24th „ „ | Shower-bath occasionally. | |
| | | | | 26th „ „ | Warm bath every night in addition. | |
| 20 | 45 | 20 | 2 grains | 10th Feb., 1855. | Admitted. | In the Asylum. |
| | | | | 4th Oct., „ | Excited; shower-bath and tart. emetic (2 grs.). | |
| | | | | 2nd Feb., 1856. | Bath and mixture continued. | |
| 21 | 46 | 20 | 1½ grains | 31st July, 1852. | Admitted. | Cured. |
| | | | | 31st „ „ | 1 gr. tart. ant. powder. | |
| | | | | 2nd Aug., „ | Repeat powder & shower-bath. | |
| | | | | 5th Oct., „ | Castor-oil and bath. | |
| 22 | About 28 | 20 | 2 grains | 27th Jan., 1849. | Admitted. | |
| | | | | 15th Sept., 1850. | Is excited. Struck the | |

| No. | Age of Patient on Entry. | Length of Bath administered, in Minutes. | When followed by Tartrate of Antimony, the Quantity. | Extracts from Case Book of Special Notes in reference to Treatment. | Result to Patient, 1856. |
|---|---|---|---|---|---|
| | | | | attendant this afternoon. Shower-bath every morning. Mix. ant. 2 oz. (2 gr. dose). | |
| | | | | June, 1851. Occasionally violent.—Sometimes has a shower-bath. | |
| | | | | 14th June, 1855. Occasionally excited and violent. *Shower-baths, with the tartar emetic mixture, are the remedies employed.* | |
| 23 | 19 | 15 or 20 | | 7th July, „ Admitted. | Cured. |
| | | | | 31st „ „ Shower-bath every morning. | |
| 24 | 37 | 15 or 20 | Not stated. | 30th „ „ Admitted. | Dead. |
| | | | | 1st Aug., „ Excited, and violent to others; shower-bath and morphia. | |
| | | | | 8th „ „ Repeat bath; emetic occasionally. | |
| 25 | 33 | 20 | Ditto | 24th Aug., „ Admitted. | Cured. |
| | | | | 8th Sept., „ Tart. ant. | |
| | | | | 22nd „ „ Shower-bath every morning, and morphia every night. | |

| No. | Age of Patient on Entry. | Length of Bath administered, in Minutes. | When followed by Tartrate of Antimony, the Quantity. | Extracts from Case Book of Special Notes in reference to Treatment. | Result to Patient, 1856. |
|---|---|---|---|---|---|
| | | | | 8th Oct., 1855. Shower-bath, tart. emetic, and blister. [*N.B. This patient imputed his recovery to the shower-bath.*] | |
| 26 | 58 | 20 | 2 grains | 29th Aug., 1855. Admitted.<br>12th Sept., „ Shower-bath.<br>20th „ „ Shower-bath, mix. tart. antimony occasionally, and blister.<br>3rd Nov., 1855. Repeat shower-bath, and hot bath at night. | |
| 27 | 39 | 20 | 2 grains | 27th Oct., 1855. Admitted.<br>4th Nov., „ He continues very excited, and is disposed to be very violent to others. Shower-bath, liquid blister, & tart. emetic mixt.<br>12th „ „ Shower-bath, with 4 t. s. of mixture occasionally.<br>15th „ „ Continue the shower-bath, with the mixture.<br>29th „ „ Shower-bath and ant. tart.<br>20th Dec., „ Ditto with morphia and ant. tart. | In house. |
| 28 | 44 | 11 | 2 grains, and a 2nd Dose. | 30th Nov., 1855. Admitted. | Cured. |

| No. | Age of Patient on Entry. | Length of Bath administered, in Minutes. | When followed by Tartrate of Antimony, the Quantity. | Extracts from Case Book of Special Notes in reference to Treatment. | Result to Patient, 1856. |
|---|---|---|---|---|---|
| | | | | 30th Dec., 1855. Made savage attacks upon his attendants. *A shower-bath and a draught containing 2 grains of tartar emetic were ordered, when he became perfectly calm and tranquil.* | |
| 29 | 25 | 20 | Nil | 5th Dec., 1855. Admitted.<br>12th „ „ Very violent. Shower-bath.<br>22nd „ „ Shower-bath occasionally. | Cured. |
| 30 | 39 | 20 | 2 grains | 21st Jan., 1856. Admitted<br>22nd „ „ Very excited. Shower-bath and acetate of morphia. *Improved from this day;* was discharged cured on 1st March, 1856. [*N.B. This patient imputed his recovery to the shower-bath.*] | Cured. |
| | 28 | 15 or 20 | Nil | 16th April, 1852. Admitted.<br>6th Oct., „ Struck the medical officer a few weeks ago for keeping him in confinement; he is sometimes abusive | In Asylum. |

| No. | Age of Patient on Entry. | Length of Bath administered, in Minutes. | When followed by Tartrate of Antimony, the Quantity. | Extracts from Case Book of Special Notes in reference to Treatment. | Result to Patient, 1856. |
|---|---|---|---|---|---|
| | | | | and excited, thereby causing a great deal of discomfort in the place. Shower-bath. | |
| 32 | about 40 | 20 | 2 grains | 3rd Sept., 1852. Admitted.<br>2nd Oct., „ Shower-bath.<br>10th March, 1853. Is in a state of lively mania, being much excited, with a disposition to mischief and violence. Shower-bath.<br>19th „ „ Continues much excited; shower-bath. Mix. ant. 2 oz. (2 gr. dose). | |

N.B.—The ages given, it will be observed, were those upon the Patients *entering* the Asylum. In many instances, therefore, an addition of several years must be made in order to show the age at date of treatment.

In looking carefully through the preceding tabulated cases, there does not appear to be one in which Mr. Snape has administered a bath of *thirty* minutes' duration. There are seventeen cases which were subjected to a twenty minutes' shower-bath; and eight who had a bath ranging from fifteen to twenty minutes. So it would appear that Dolley was the only patient in the asylum who had a *thirty* minutes' bath! Mr. Snape offers no explanation why he went, *per saltum*, from *twenty* to *thirty* minutes in this single case. It is clearly the first one in the records of the asylum in which a prolonged shower-bath of thirty minutes' duration was ordered. If there had been other cases of the kind, Mr. Snape certainly would have embodied the particulars in the tabular statement which he has put forward in his defence. But let us, for a few minutes, analyse these tables. Case No. 1 had a bath of "fifteen *or* twenty minutes," with one and a-half and two grains of tartrate of antimony. No result specified. Case 2—treatment the same: no result stated. Case 3—a bath of twenty minutes, with emetic mixture. Result—"Found beneficial." Case 4—a bath of fifteen *or* twenty minutes, with the emetic mixture. Result—"Prescribed with advantage." Case 5—similar treatment. Result—"Cured." Case 6—similar treatment. This patient appears to have been under treatment from the 2nd of February, 1852, and to have remained in the asylum until the 23rd of February, 1855, when it is alleged he was discharged "cured," by means of the shower-bath and emetic mixture. It is useless to trouble our readers with any further analysis; the tables are before them, and they can read and judge for themselves. It is, however, clearly apparent that the shower-bath and emetic mixture were almost invariably exhibited when the patient committed an act of insubordination, and was said to be violent, noisy, and destructive; leading one to the conclusion that these depressing agents were mainly employed to induce, on the part of the patient, docility and quietness. Whenever a patient is "noisy and violent," "breaks windows," exhibits "a disposition to violence," "disposed to be mischievous and violent to others," "attacks those around him with a knife," "abusive, and destructive of his clothing," &c. &c., he is immediately cooled down by means of the prolonged shower-bath and the "white-coloured mixture," *alias*, tartar emetic! Is this not an unjustifiable abuse of an alleged valuable curative agent? In cases like those previously cited, Mr. Snape's difficulty will be to persuade the public that the baths were not used as a *quasi*-punishment. It is clear by his own admissions that he has exposed himself to such an imputation. In the inquiry before the Commissioners in Lunacy, the following questions were put to Dr. Diamond:—

"*Q.* Did you ever order a shower-bath in the Male Ward when that department has been under your care ?

"*A.* Yes. I have once, and that has been in Mr. Snape's absence. One of his patients knocked out a tooth of one of my patients, and was guilty of violence.

It would appear by the above extract from Dr. Diamond's evidence, that one of Mr. Snape's patients, in a personal conflict, knocked out the tooth of one of his patients, and was subjected to a shower-bath ! In justice to himself, Dr. Diamond should explain whether he ordered the bath as a punishment, or as a remedial agent ? The facts cited by Mr. Snape, fully justify the suspicion that this highly eulogised mode of treatment has not altogether been confined to its strictly medical and legitimate uses. We cannot too strongly repudiate the idea of inflicting *punishment* upon the insane, in or out of an asylum. It should never be forgotten that the inmates of a lunatic asylum are *irresponsible*, particularly for actions committed in moments of maniacal and delirious excitement, and whilst under the influence of strong delusions.*

One word as to the duration of these much-vaunted baths. Many of the cases referred to in Mr. Snape's tables, were said to have had a shower-bath of "fifteen *or* twenty minutes'" length. How is it that the *exact duration* of the bath is not stated ? Would it not appear that no accurate computation of time was made when the shower-baths were used ? A patient is ordered to the bath,—does the attendant stand by its side, with his watch in his hand, to measure the time? If not, how easily may a man with the most honest intentions be deceived if he "guesses" at the duration of the bath. In *eight* of the cases referred to by Mr. Snape the time could not have been accurately computed ; for the patients are said to have been in the bath "fifteen *or* twenty minutes."

May not the same inaccurate measurement of time have crept into the other cases, where the period is positively stated ? The matter is clearly open to grave suspicion. The attendants employed to administer the baths, may unintentionally have deceived Mr. Snape as to their duration ; until we are satisfied as to the time being carefully computed, no conclusions as to the safety or efficacy of prolonged shower-baths can be safely drawn.†

* Let our readers imagine the following extracts from the daily case-book of a County Lunatic Asylum. A. B.—Violent ; broke six panes of glass. Ordered eight leeches to the head. F. G.—Kicked one of the attendants. A blister to be applied to the nape of the neck ; to be dressed, as an additional punishment, with turpentine ointment. G. W.—Tore his clothes. An emetic was administered. F. H.—Soiled his bed. Ordered to be cupped. P. T.—Shook his fist in the face of the medical superintendent, and used threatening language. Calomel and jalap to be given. A. J.—Destroyed his bed-clothes. Ordered a good dose of opium !

† Mr. Snape, with the view of establishing the perfect safety of the shower-bath

It appears that Dr. French, who officiated for Mr. Snape during his suspension, did not, on his first entering upon the performance of his duties, use the shower-bath at all. Mr. Snape says:—

"It appears that Dr. French, when first appointed, alarmed very naturally by my then position before the public, wholly refrained from using the shower-bath from the date of his appointment to the 14th of August last, a period of three months, *but used the plunge-bath in its stead.*"

The plunge-bath in lieu of the shower-bath!* The old story of *scylla* and *charybdis*. Have our readers any idea of what a plunge-bath is? If not, we will enlighten them. A violent and excited patient is taken forcibly by his legs, and plunged headforemost into an ordinary swimming-bath. He is not permitted the use of his limbs when in the water, but is detained there, or taken out and plunged again into the bath, until the *required effect* (?) is produced! Of course, quietness, submission to authority, docility, and freedom from excitement and violence, are the natural consequences of this *gentle* and *soothing* mode of treatment. Is it possible that in the present enlightened age an obsolete and exploded process of cure (?) like this should have been revived in one of our large county lunatic asylums, and that, too, in an establishment whose principal officer was under a cloud for an alleged abuse of prolonged shower-baths?

It would give us pain if anything that we have said in the preceding pages respecting the use of the prolonged shower-bath, were construed to the injury or personal disadvantage of Mr. Snape. We have always heard him spoken of as a humane man, anxious to perform every duty of life honourably and conscientiously. He has committed a mistake on this *one* point, and the best among us are not free from error.

Having been restored to an important official position as the medical superintendent of the male department of the Surrey County Lunatic Asylum; being intrusted with the lives, as well as with the care and treatment, of a large number of the most used in the Surrey Asylum, exposed himself to its influence for a period of three-quarters of an hour; and, we are glad to report, escaped unscathed! This proves nothing. Mr. Snape is a healthy man; he went into the bath *voluntarily*, with, we presume, a determination to pass safely through the trying ordeal. How different to the case of a man suffering from a depressing disease, connected with an affection of the brain, or a shattered nervous system, forced into this bath, and detained there against his will! Mr. Snape does not tell us what he took before going into the bath, or what he administered to himself after he came out of it. We will venture to say that he did not take a dose of the white-coloured mixture.

* We have no hesitation in speaking in terms of unqualified disapprobation of the plunge-bath as a method of treatment, punishment, or discipline. Is it true that the plunge-bath is used as a means of treatment at St. Luke's Hospital?

dejected and helpless of the severely-afflicted family of man,—we have, occupying as conductor of this Journal a position of grave responsibility as a public instructor, conceived it to be our painful duty to discuss with considerable latitude, and unreserved and unrestricted freedom, the specific mode of treatment suggested by this gentleman for the treatment of the insane. We have spoken of the prolonged shower-bath and the tartar-emetic in terms of severity, but, we unhesitatingly maintain, in the strictest accordance with truth and justice. We have no desire to injure or disparage Mr. Snape, whilst exercising our undoubted editorial prerogative of freely criticising the principle of treatment he has recommended for our adoption.

"*Licuit, semperque licebit,*

*Parce personis, discere de Vitiis.*"

